# *De novo* assembly of the pepper transcriptome (*Capsicum annuum*): a benchmark for *in silico* discovery of SNPs, SSRs and candidate genes

**DOI:** 10.1186/1471-2164-13-571

**Published:** 2012-10-30

**Authors:** Hamid Ashrafi, Theresa Hill, Kevin Stoffel, Alexander Kozik, JiQiang Yao, Sebastian Reyes Chin-Wo, Allen Van Deynze

**Affiliations:** 1 Seed Biotechnology Center, University of California, Davis, 1 Shields Ave, Davis, CA 95616, USA; 2 Genome Center, University of California, Davis, 1 Shields Ave, Davis, CA, 95616, USA; 3 Present address: Interdisciplinary Center for Biotechnology Research, University of Florida, Gainesville, FL, 32610, USA

**Keywords:** Pepper, Capsicum spp, Molecular Markers, EST, Transcriptome, RNAseq, Annotation, SNP, SSR, SPP

## Abstract

**Background:**

Molecular breeding of pepper (*Capsicum spp.*) can be accelerated by developing DNA markers associated with transcriptomes in breeding germplasm. Before the advent of next generation sequencing (NGS) technologies, the majority of sequencing data were generated by the Sanger sequencing method. By leveraging Sanger EST data, we have generated a wealth of genetic information for pepper including thousands of SNPs and Single Position Polymorphic (SPP) markers. To complement and enhance these resources, we applied NGS to three pepper genotypes: Maor, Early Jalapeño and Criollo de Morelos-334 (CM334) to identify SNPs and SSRs in the assembly of these three genotypes.

**Results:**

Two pepper transcriptome assemblies were developed with different purposes. The first reference sequence, assembled by CAP3 software, comprises 31,196 contigs from >125,000 Sanger-EST sequences that were mainly derived from a Korean F_1_-hybrid line, Bukang. Overlapping probes were designed for 30,815 unigenes to construct a pepper Affymetrix GeneChip® microarray for whole genome analyses. In addition, custom Python scripts were used to identify 4,236 SNPs in contigs of the assembly. A total of 2,489 simple sequence repeats (SSRs) were identified from the assembly, and primers were designed for the SSRs. Annotation of contigs using Blast2GO software resulted in information for 60% of the unigenes in the assembly. The second transcriptome assembly was constructed from more than 200 million Illumina Genome Analyzer II reads (80–120 nt) using a combination of Velvet, CLC workbench and CAP3 software packages. BWA, SAMtools and in-house Perl scripts were used to identify SNPs among three pepper genotypes. The SNPs were filtered to be at least 50 bp from any intron-exon junctions as well as flanking SNPs. More than 22,000 high-quality putative SNPs were identified. Using the MISA software, 10,398 SSR markers were also identified within the Illumina transcriptome assembly and primers were designed for the identified markers. The assembly was annotated by Blast2GO and 14,740 (12%) of annotated contigs were associated with functional proteins.

**Conclusions:**

Before availability of pepper genome sequence, assembling transcriptomes of this economically important crop was required to generate thousands of high-quality molecular markers that could be used in breeding programs. In order to have a better understanding of the assembled sequences and to identify candidate genes underlying QTLs, we annotated the contigs of Sanger-EST and Illumina transcriptome assemblies. These and other information have been curated in a database that we have dedicated for pepper project.

## Background

Pepper (*Capsicum spp.*) is a member of the Solanaceae family which is becoming an increasingly important vegetable crop worldwide due to its wide diversity and high quality in flavor, concentration of vitamins and other antioxidants. In addition to its dietary and culinary importance, capsaicinoid compounds of pepper are being used in the pharmaceutical industry [1]. Pepper has five domesticated and 15–20 wild crossable species. Similar to other members of the Solanaceae family such as tomato, pepper has been used as a model organism for classical and molecular genetics analyses. Selfing and crosses among and within certain species can be made readily and multiple generations can be easily produced each year. This creates the genetic diversity that is required for conventional breeding programs. However, the commercial application of these genomic resources for gene discovery and molecular breeding has been limited by paucity of available informative molecular markers. The limited amount of molecular markers is primarily due to lack of availability of the pepper genome sequence and sequence resources. For instance, over the past two decades, a variety of molecular markers (AFLP, RFLP, SSR, COSII and RAPD) have been developed and applied to several intra- and inter-specific crosses of pepper [2-4]. However, due to their nature, the majority of these markers, except COSII and tomato cDNA markers, that have been used in the published genetic maps of pepper are not high throughput or gene-based.

From among many types of molecular markers that have been developed during the past three decades, Simple Sequence Repeats (SSRs) and Single Nucleotide Polymorphisms (SNPs) are the most attractive ones for breeding [5]. SSRs are co-dominant and can be assayed in any laboratory with minimum facilities or they can be automated with capillary sequencers for moderate throughput. On the other hand, SNPs are extremely abundant; the majority are biallelic; they are easily scored and can be tightly linked to or are the actual cause of allelic (phenotypic) differences in traits. Moreover, there are several high-throughput technologies based on allele-specific PCR, hybridization and single base-pair extension which makes them cost-effective for assaying large numbers of genotypes once robust SNPs have been identified. However, without using recent bioinformatics tools and next generation sequencing (NGS), identifying SNPs and SSRs within a genome as large as pepper (3.5 Gb) [6] is not a trivial task.

Prior to the advent of NGS technologies, discovering putative SNPs was achieved using low throughput electrophoresis or capillary sequencing [7,8]. These methods are lengthy, low coverage and expensive per data point. However, we should recognize that Sanger sequencing has provided a wealth of EST sequences that have been the primary basis of identifying SNPs [9]. In 2006 at the onset of the Pepper GeneChip® project the sequencing resources of pepper were largely limited to the EST sequences that were developed by Dr. Doil Choi at Seoul National University. Assembling the EST sequences into unigenes and mining SNPs *in silico* is one of the approaches that has been used for marker development [7,10]. In order to take the genotyping resources of pepper to the next level- we used Affymetrix GeneChip arrays [11,12] as a new tool for massively parallel marker discovery and genotyping in pepper. This novel tool uses a new generation of markers called Single Position Polymorphisms or SPPs [12]. Therefore, assembling ESTs enabled us, first to design and produce the genotyping chips and second to extract a wealth of polymorphism in pepper.

In recent years, sequencing of expressed genes (transcriptomes) using NGS technologies such as SOLiD, Illumina and 454, has been used for gene discovery and allele mining [13-15]. This method, also known as RNAseq, has been used in many plant and animal species such as maize [13], brassica [14], Arabidopsis [16], rice [17], human [18], and mouse [19]. With the advent of NGS technologies, the number of publications describing *de novo* assemblies of plants transcripts and other organisms has been increasing constantly. In addition to availability of sequences, bioinformatics tools have also been developed to process, analyze and store the massive data that are generated daily. For instance, one of the most popular assemblers is the Velvet [20] software package which is able to assemble short reads derived from Illumina into contigs using de Bruijn graphs [20] algorithm. However, Velvet is not the only assembler for short reads. SOAP *de novo*, ABySS [21] and CLC Genomics Workbench, which is commercially available, are just a few examples of many other assemblers. In the current study we took advantage of both Velvet and CLC to make *de novo* assemblies of transcriptomes of three pepper lines, Maor, Early Jalapeño and CM334.

The final goal in many transcriptome sequencing efforts is to annotate sequences by connecting them to biological information. Annotation of sequences allows one to have insight into the function and structure of the genome. Without annotation, sequences have little meaning. Availability of intronic regions through genome sequencing facilitates gene model predictions, which help to identify locations of regulatory elements as well as alternate splicing events. However, for pepper, a whole genome sequence is still not available and to-date all annotations have been carried out on transcriptome sequences [22-24]. Automated annotation is an approach that provides us an immediate response to a question that we pose. Is there any similarity between unknown sequences and previously characterized sequences from the same or other species? Normally this will be done by the basic local alignment search tool (BLAST) to find the best matches between the unknown and known sequences followed by mapping the results to Gene Ontology (GO) terms [25] and associating the GO terms with functional proteins, using the results of previous steps. In the present study we performed an *in silico* annotation of both Sanger-EST and IGA transcriptome assemblies of pepper. The current annotation information can be used for candidate gene discovery, identification of regulatory elements and gene prediction before the full annotation of a pepper genome becomes available. We have also developed a MySQL database and a web interface that can be queried to find information about the assemblies, such as SSR or SNP makers within each contig and to find their corresponding annotation.

## Results

### Pepper Sanger-ESTs assembly

**Figure 1:**
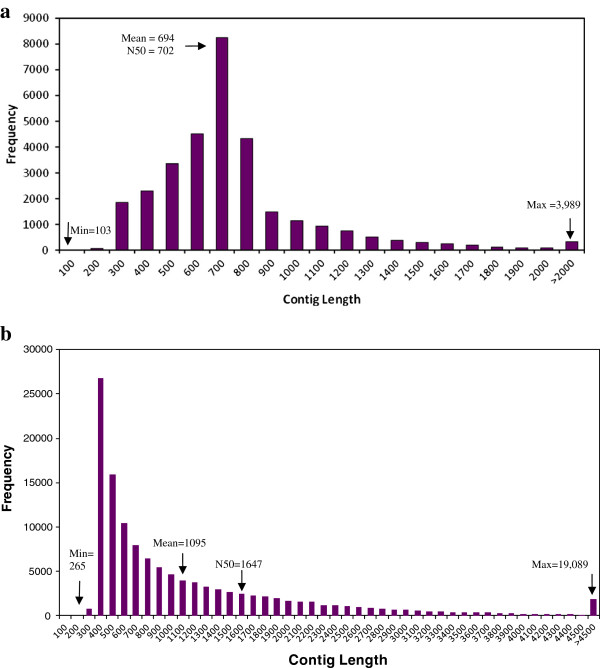
a) Distribution of contigs length in a) pepper Sanger-EST assembly b) distribution of contigs length in pepper IGA transcriptome assembly.

We developed a non-redundant set of unigenes based on all available sequences for pepper (in 2006) to design a tiling Affymetrix GeneChip array for marker discovery and application in pepper [11]. Merging the KRIBB (see list of URLs) sequences (115,787) with the processed GenBank sequences (9,905) resulted in 125,692 sequences. After trimming, a total of 123,489 sequences remained, including 121,867 EST sequences, 515 assembled mRNAs, 465 genomic sequences and 642 COSII marker sequences (Table 1). *C. annuum* made up 99.5% of the sequences with minor representation from, *C. frutescens, C. chinense* and *C. baccatum*. Hereafter, the assembly of Sanger ESTs is called the Sanger-EST assembly. In the Sanger-EST assembly, 32,071 unigenes were obtained with 12,970 consensus sequences and 19,101 singletons. The number of unigenes account for 25.8% of initial input sequences (123,489). Unigenes with a size less than 200 nucleotides (nt) accounted for 2.7% of the total unigenes. The summary statistics of the Sanger-EST assembly are presented in Figure 1a and Table 2. The final assembly, consisting of 31,196 unigenes greater than 200 nt, was annotated and mined for SSRs and SNPs.

**Table 1:** A summary of sequences included in the pepper Sanger-EST assembly

**Sequence Type/Species**	**No. of Sequences Before Filtering**	**Total on the Chip**
**ESTs**		123,489
*C. annuum*	125,320	
*C. chinense*	372	
**Genomic**		465
*C. annuum*	318	
*C. baccatum*	28	
*C. chinense*	31	
*C. frutescens*	27	
Others	61	
**Annotated mRNA**		515
*C. annuum*	427	
*C. chinense*	76	
Others	12	
**COSII**	642	642

**Table 2:** Comparison of assembly of pepper Sanger ESTs versus assembly of IGA reads

**Statistics**	**EST Assembly**	**Transcriptome Assembly**
Number of Unigenes	31,196	123,261
Total assembled nucleotides	21,665,127	135,019,787
Average GC content (%)	41	39
Longest Contig Length	3,989	19,089
Average Contig Length	694.5	1,095
Median Contig Length	651	697
N50	702	1,647
Number/Percent Contigs Size < 1 KB	27,248	78,433
1–2 KB	3,634	27,436
2–3 KB	288	10,616
3–4 KB	26	3,955
4–5 KB	0	1,559
5–10 KB	0	1,184
10–20 KB	0	78

### *De novo* pepper Illumina transcriptome assembly

The Illumina transcriptome sequencing generated ~53 M, 57 M and 90 M cleaned and trimmed reads in CM334, Maor and Early Jalapeño, respectively. The raw data were submitted to NCBI Sequence Read Archive (SRA) under accession No. SRA052314.2 and the trimmed reads submitted to European Nucleotide Archive (ENA) under study number ERP001411. The more stringently trimmed reads ranged between 25 to 70 nt in length as described in methods. To compare assembly performance of different *k-mer* (see below for definition) values [20], we tested *k* values of 31, 35 and 41 bp. Applying different *k-mers* resulted in the use of different numbers of reads but the overall trend was toward the use of more reads in the assembly as the *k-mer* increased from 31 to 41. In Velvet, 64% - 79% of the sequences were used in each assembly as the *k-mer* value was increased. Both Velvet and CLC produced significantly fewer contigs, with average reductions ranging from 48% in Velvet to 35% in CLC, when using stringently trimmed data. For instance, in the case of Early Jalapeño by using untrimmed and trimmed data at *k* = 31 bp, the number of contigs generated in the two assemblies was 68,737 and 39,956, respectively. The fraction of contigs longer than 1 KB varied from 83% (*k* = 31 bp) to 72% (*k* = 31) for untrimmed and trimmed data (Table 3). Median weighted (N50) lengths of assemblies were highest at *k* = 41 bp for both untrimmed and trimmed data (Table 3). The meta assembly which is called hereafter the pepper IGA (Illumina Genome Analyzer) transcriptome assembly, comprises assembly of contigs from Velvet and CLC and had the largest median of all assemblies (N50=1,647) with 123,261 contigs and an assembly of >135M bases (Figure 1b and Table 2). The final results and steps to generate *de novo* assembly of pepper IGA reads are presented in Table 4.

**Table 3:** The effect of trimming the reads and k-mer length on the number of contigs and N50 in IGA transcriptome assembly

Type of Reads/k-mer value^*^	**CM334**				**Maor**				**Early Jalapeño**			
	**Total No. of Contigs**	**No. Contigs >1 KB**	**Percent Contigs > 1 KB**	**N50**	**Total No. of Contigs**	**No. Contigs > 1 KB**	**Percent Contigs > 1 KB**	**N50**	**Total No. of Contigs**	**No. Contigs > 1 KB**	**Percent Contigs > 1 KB**	**N50**
Untrimmed/K31	65,337	52,179	80	603	62,570	50,306	80	589	68,737	57,077	83	497
Untrimmed/K35	64,096	49,875	78	680	61,561	48,345	79	660	68,237	55,564	81	562
Untrimmed/K41	52,099	36,770	71	947	50,290	35,900	71	926	58,431	44,045	75	777
Trimmed 25–70/K31	42,310	30,628	72	864	36,173	24,871	69	995	39,956	28,711	72	870
Trimmed 25–70/K35	34,525	22,627	66	1,109	30,202	18,859	62	1,205	34,497	23,039	67	1,057
Trimmed 25–70/K41	27,439	16,728	61	1,239	26,885	16,660	62	1223	28,588	18,162	64	1,165

* The untrimmed reads were between 40–80 nt long. The same reads were trimmed by 5 and 10 nt from 5' an 3' ends respectively to eliminate the possible sequencing errors at the beginning and at the end of each read. The numbers were selected arbitrarily but K=31 is the Velvet recommended value.

**Table 4:** Summary statistics of transcriptome assembly of three pepper lines using Velvet, CLC and CAP3 assemblers

**Name of Assembly**	**Assembler**	**CM334**			**Maor**			**Early Jalapeño (EJ)**		
		**No. of Contigs**^**a**^	**N50**	**Total Assembled nt.**	**No. of Contigs**^**a**^	**N50**	**Total Assembled nt.**	**No. of Contigs**^**a**^	**N50**	**Total Assembled nt.**
Super Assembly	Velvet^b^	75,853	1,287	71,903,681	70,459	1,303	67,210,074	81,973	1,198	73,865,962
	CLC^c^	83,187	1,357	79,564,926	76,542	1,389	74,367,265	81,528	1,347	78,144,374
Mega Assembly	CAP3	83,113	1,488	84,792,180	76,375	1,526	79,383,673	82,614	1,488	84,973,865
(Velvet+CLC)								**Combined CM334, Maor, EJ**		
Meta Assembly (3 lines)	CAP3							123,261	1,647	135,019,787

^a^ No of contigs longer than 300 nucleotides were included in all assemblies.

^b^ Six Velvet iterations including three k-mers of normally trimmed data and 3 k-mers of stringently trimmed data were assembled using CAP3 program.

^c^ Two CLC iterations including one normally trimmed data and one stringently trimmed data were assembled using CAP3 program.

### Annotation of Sanger-EST assembly

**Figure 2:**
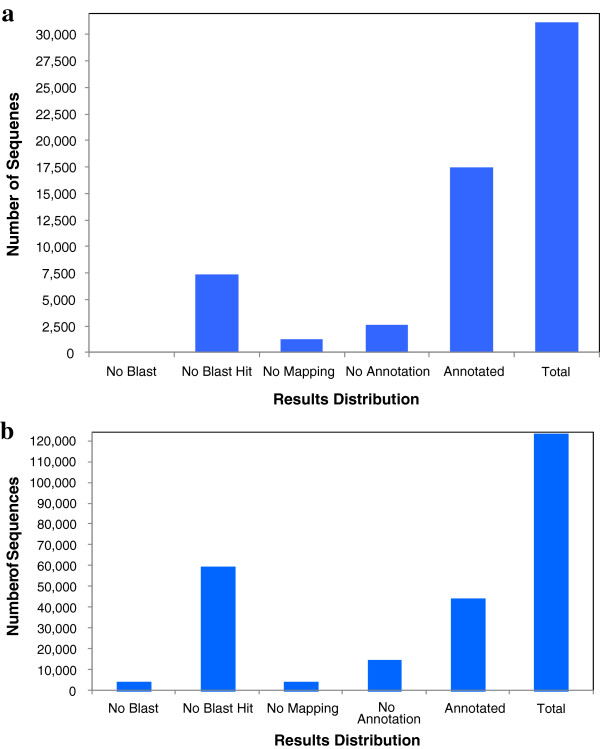
Distribution of Blast2GO three-step processes including BLASTX, mapping and annotation of for a) Sanger-EST assembly and b) IGA transcriptome assembly.

**Figure 3:**
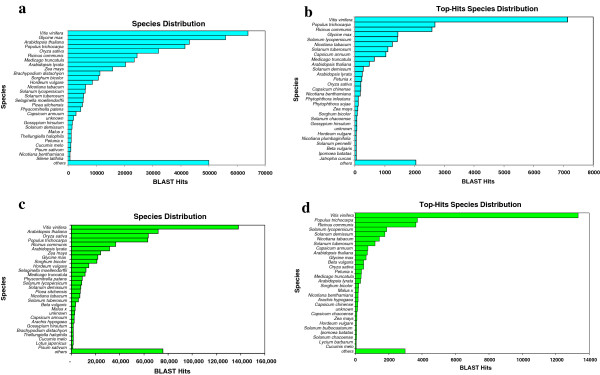
**a) Species distribution by accounting all BLASTX hits in the Sanger-EST assembly b) Top-hit species distribution based on BLASTX alignments in the Sanger-EST assembly. c) Species distribution by accounting all BLASTX hits in the transcriptome assembly d) Top-hit species distribution based on BLASTX alignments in the IGA transcriptome assembly**. Cultivated *Solanum* species are more frequent than wild type species (*S. habrochaites* or *S. bulbocastanum*). Within *Capsicum* species, there are more hits to *C. annuum* than *C. chinense* or other distantly related capsicum species such as *C. chacoense.*

Both assemblies were annotated using Blast2GO software [26]. Blast2GO annotation is Gene Ontology (GO) based data mining for sequences with unknown function [25,26]. The results of each step of Blast2GO annotation of the Sanger-EST assembly are summarized in Figure 2a. BLASTX of the Sanger-EST assembly unigenes against the GenBank non-redundant protein database resulted in the identification of 24,003 (76.9%) sequences with at least one significant alignment to an existing gene model and with an average contig length of 745 nt. These contigs covered 21.6M bases (82.5%) of the total Sanger-EST assembly. The 7,193 unigenes that did not have any hit in the GenBank were on average 525 nt long and were covering 3.8M (17.5%) bases. The mapping step of Blast2GO resulted in association of 22,728 (72.8%) unigenes with GO terms [25] (Figure 2a). The unigenes were assigned between 1 and 50 GO terms with a weighted average of five GO terms per unigenes. The annotation step of Blast2GO assigned functions to 18,715 (60%) of unigenes. A query with InterProScan increased the number of annotated unigenes by 17%. The results of the Blast2GO annotation were merged with the results of the InterPro annotation to maximize the number of annotated sequences. By categorizing all BLASTX results, *Vitis vinifera*, *Glycine max*, *Arabidopsis thaliana, Populus trichocarpa* and *Oryza sativa* were among the top five plant species in terms of the total number of hits to the Sanger-EST unigenes (Figure 3a). However, when the results were categorized based on the highest similarity between each of the Sanger-EST unigenes and sequences in the databases, the top five plant species were *V. vinifera*, *P. trichocarpa*, *Ricinus communis*, *G. max and Solanum lycopersicum* (Figure 3b). Direct GO count graphs were created to categorize the sequences to several groups based on their biological processes, molecular functions and cellular component ontologies. Inside the biological processes category, sequences in cellular process, metabolic process, response to stimulus, biological regulation and localization had the highest frequencies. In terms of molecular function, transferase activity, nucleotide binding and ion binding related sequences were the top three GO terms in the Sanger-EST assembly. Among cellular components, the GO terms corresponding to constituents of the cytosol (intracellular fluid), intracellular part (any constituent part of the living contents of a cell), plasma membrane and organelle had the highest numbers in the assembly [See Additional File 1: Figure S11a-c]. The results of annotation can be accessed and queried through the pepper GeneChip database [See list of URLs] or [See Additional File 2: Table S1].

### Annotation of IGA transcriptome assembly

**Figure 4:**
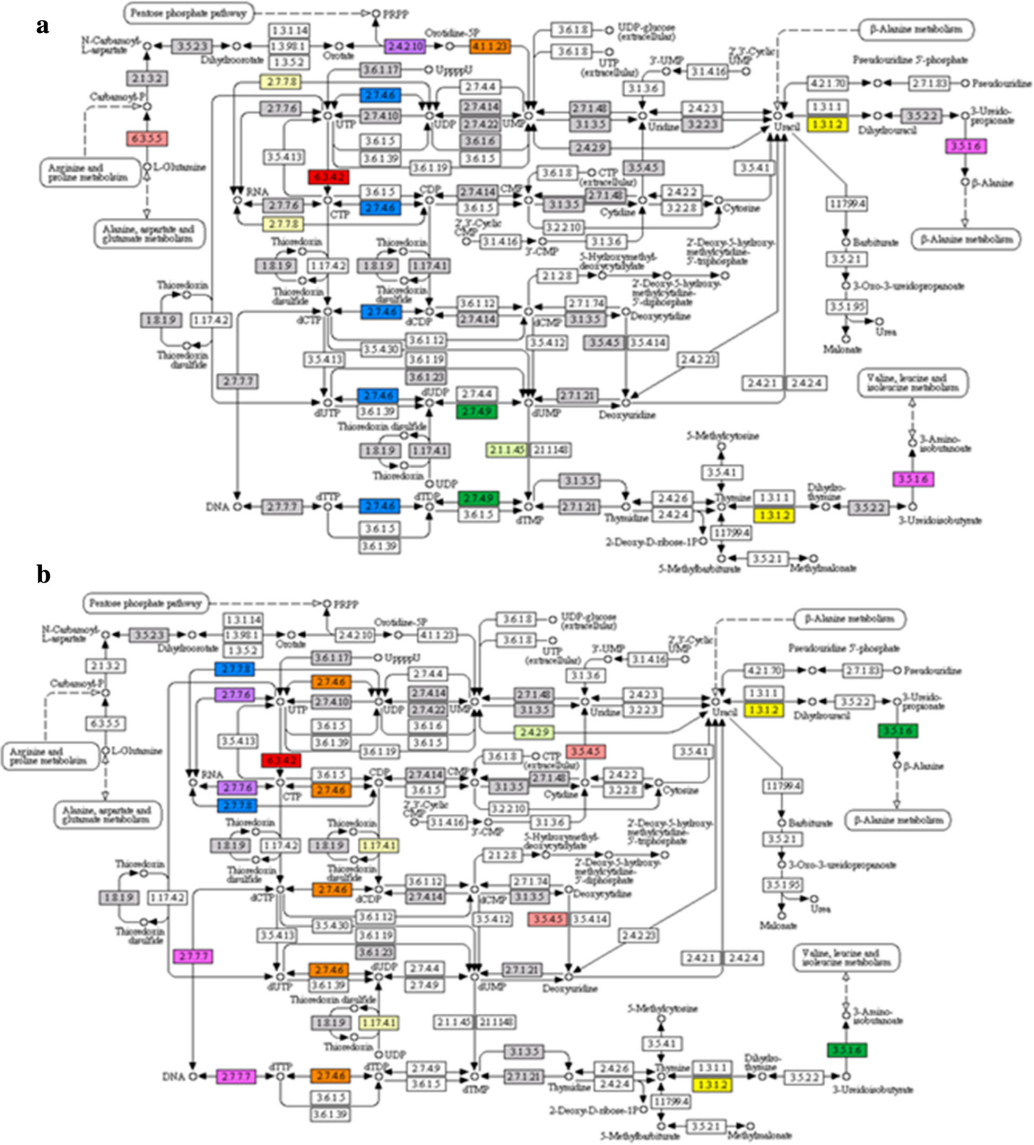
**An instance of a KEGG map for Pyrimidine metabolism pathway**. Each box represents the enzyme code involved in each section of the pathway. The colored boxes are depicting identified enzymes by **a**) Sanger-EST assembly and **b**) transcriptome assembly. The KEGG files can be downloaded from Pepper GeneChip website (https://pepper.ucdavis.edu).

The three steps of Blast2GO annotation of the IGA transcriptome assembly are summarized in Figure 2b. A total of 63,202 contigs (51.3%) with an average length of 1,495 nucleotides had at least one significant alignment with a protein in the non-redundant database of GenBank. These contigs covered 94.5M bases, (70%) of the total assembly. However, the 60,055 (48.7%) contigs that did not have hit to any sequence in GenBank were on average 674 nucleotide long and covered 40.5 M bases, (30%) of the total assembly. The mapping step of Blast2GO identified 37,918 (30.7%) contigs with GO terms. A significant amount of mapping data (91.5% of contigs with mapping information) were derived from UniProtKB database followed by TAIR and GR_protein. In addition, 13 other databases were searched but did not significantly contribute to the mapping process. Between 1–80 GO terms were assigned per sequence with a weighted average of 5 GO terms per contig (Figure 2b). Twelve percent, (14,740) of contigs, were annotated as functional proteins. The frequency of GO terms for shorter sequences (<2.5KB) was less than that of longer sequences. The percentage of annotated sequences increased proportionally with their length, such that sequences longer than 4.8 KB were 100% annotated. As expected, the majority of annotations were inferred electronically compared to direct assays (14%) [See Additional File 1: Figure S5–S6]. By counting all significant hits in the BLASTX result table, *V. vinifera*, *A. thaliana* and *O. sativa* were the top three species in terms of hit number (Figure 3c). As Figure 4c depicts, based on this grouping *Solanum sp.* did not have as many hits as other less closely related species to pepper. However, when we filtered the BLASTX results based on similarity of pepper contigs with Solanum species, *Solanum sp.* were ranked after *V. vinifera*, *P. trichocarpa* and *R. communis* (Figure 3d). InterProScan, Annex, and GO annotation query through more than 16 databases significantly increased annotation by 15%. Direct GO count graphs were created to categorize the sequences based on their biological processes and molecular functions as well as their cellular component. Based on their biological processes sequences involved in cellular process, metabolic process and response to stimulus had the maximum frequencies. In terms of molecular function, nucleic acid binding elements comprised the highest numbers of sequences in the IGA transcriptome assembly, followed by transferase activity and nucleotide binding related sequences. Cellular component constituents of intracellular organelle, cytoplasm and cytoplasmic part and plasma membrane were among sequences that had the maximum numbers in the assembly [See Additional File 1: Figure S12a-c]. The KEGG maps for more than 130 metabolic pathways were generated for both assemblies and the results were exported. Two examples of KEGG maps for the Pyrimidine metabolism pathway are depicted in Figure 4a-b. The KEGG map files, the Blast2GO project files (.dat files), InterProScan and BLASTX files are available to download at the Pepper GeneChip website. The results of annotation also can be accessed through the Pepper GeneChip database or [See Additional File 2: Table S2].

### SSR discovery in the Sanger-EST and the IGA transcriptome assemblies

From the 31,196 unigenes (21.6 M bp) in the Sanger-EST assembly, 2,357 (7.5%) unigenes contain putative SSRs, from which 253 unigenes bear more than one SSR marker signature. A total of 2,489 SSRs with simple repeats and 183 (~7%) SSRs with compound formation were identified. From 123,261 contigs that were examined in the IGA transcriptome assembly, 9,498 contigs were identified with 10,396 SSRs of which 617 (5.6%) SSRs were of compound formation. From 9,498 SSR-containing contigs, 1,236 (13.0%) had more than one SSR sequence. Using Primer3 software we were able to design primers for 1,533 and 7,458 putative SSR markers in the Sanger-EST and the IGA assemblies, respectively. A total of 859 SSRs were identified with identical motif and size between the two assemblies, resulting in 8,132 unique SSRs. In both assemblies, di-nucleotide AG/CT was the most frequent SSR motif followed by AC/GT or AT/TA. The tri-nucleotide motif AAC/GTT was more frequent in the IGA transcriptome assembly than that of the Sanger-EST assembly, while AAG/CTT was more frequent in the Sanger-EST assembly than the IGA transcriptome assembly. Overall, tri-nucleotide motifs were more frequent in our IGA transcriptome assembly than the Sanger-EST assembly. Longer motifs such as tetra and penta-nucleotide motifs were less frequent than di- and tri- nucleotide motifs [See Additional File 3: Tables S3 and S6]. Additional File 3: Tables S4 and S7 provide lists of SSRs in the Sanger-EST and the IGA assemblies, respectively. Where possible the primers were designed for SSRs in both assemblies [See Additional File 3: Tables S5 and S8] and common SSRs are listed in Additional File 3: Table S9.

### SNP discovery in Sanger-EST assembly

A majority of ESTs that were used in the Sanger-EST assembly were obtained from cDNA libraries of a Korean F_1_ hybrid (Bukang). Accordingly, most of the SNPs that we identified would belong to polymorphism between the parents of this particular F_1_ individual. In the Sanger-EST assembly we had 12,970 unigenes that resulted from greater than one EST. The remaining unigenes were single sequences that could not be examined for presence of SNPs. Analysis of 12,970 unigenes resulted in identification of 4,234 putative SNPs from 1,854 contigs [See Additional File 3: Table S10], an average of 0.3 SNPs per contig. The 12,970 contigs comprise 11,847 KB of pepper Sanger-EST assembly. Therefore, on average 1 SNP per 2,798 bases of pepper Sanger-EST assembly was identified.

### SNP discovery in the IGA transcriptome assembly

The IGA transcriptome assembly comprised 123,261 unigenes including 48,642 contigs (assembled contigs of three genotypes) and 74,619 singletons (assembled sequences from a single genotype). In order to make a fair comparison between the IGA transcriptome assembly and the Sanger-EST assembly, we employed only 48,642 contigs of the IGA transcriptome assembly to discover SNPs. Using only contigs in the assembly a total of 47,686 putative SNPs were identified, that is ~1 SNP per contig. SNPs were filtered for the presence of an adjacent SNP in the vicinity of 50 bases. After filtering, a total of 30,495 SNPs were remaining that were used in calculation of SNP density. The 48,642 contigs that were mined for SNPs represented 76,952 KB of the pepper IGA transcriptomes that contains on average 1 SNP per 2,523 bases identified in the pepper IGA transcriptome assembly.

In order to provide a set of more reliable set of putative SNPs to the public; in a separate analysis all 123,261 sequences of IGA transcriptome assembly were used, thereby a total of 51,029 putative SNPs were identified. These putative SNPs were first filtered for the presence of flanking intronic region junction, adjacent putative SNPs as well as heterozygote positions in their 50 bp vicinity (See Materials and Methods). After applying all of the filters across the three genotypes used for the IGA transcriptome assembly, a total of 22,863 putative SNPs were retained [See Additional File 3: Table S11]. The SNPs were submitted to Database of Single Nucleotide Polymorphisms (dbSNP). Bethesda (MD): National Center for Biotechnology Information, National Library of Medicine. dbSNP accession:[ss523750580 – ss524344808], (dbSNP Build ID: B138), available from NCBI SNP database website.

### Comparison of SNPs between the Sanger-EST and the IGA transcriptome assemblies

To identify the unique and common SNPs between the two assemblies, the following alignments were made reciprocally for each SNP using BLASTN. A 101 nt fragment from the Sanger-EST assembly with a SNP at the center was aligned with all SNP-containing fragments (101 nt) of the IGA transcriptome assembly and vice versa. A valid hit counted as the one with a minimum of 80 nt matches in the alignment. The reciprocal comparison of SNPs from each assembly determined that 3,918 out of 4,235 SNPs were unique to the Sanger-EST assembly [See Additional File 3: Table S12]. Of the 22,863 SNPs from the IGA transcriptome assembly 22,548 (98.6%) SNPs were unique to that assembly [See Additional File 3: Table S13]. Finally, a total of 316 common SNPs between the two assemblies were identified by this analysis [See Additional File 3: Table S14] resulting in 26,782 unique SNPs.

### SNP validation

Out of 142 SNPs assayed, three (2.1%) did not produce any PCR product and 13 (9.1%) had ambiguous calls, that is one allele was called correctly according to our SNP discovery pipeline but the alternate allele could not be unequivocally determined by KASPar assay. Out of 126 remaining SNP assays [88 Sanger-EST assembly SNPs and 38 verified by SPPs (See Stoffel et al. for definition)], 113 (89.7%) were polymorphic (at least one of the two alleles was observed across 47 genotypes) and 13 were monomorphic across the genotyping panel (FDR=10%). From 113 polymorphic assays, 78 (70%) and 35 (30%) were SNPs and SPP, respectively. Therefore, 78 out of 88 (89%) amplifiable SNP assays were polymorphic across the diversity panel described in Hill et al. [11] [See Additional File 3: Table S15]. We also investigated the polymorphism rate of 78 putative SNPs among the three pepper genotypes that were used for transcriptome assembly (CM334, EJ and Maor). A total of 40 out of 78 (51%) assays were polymorphic in the diversity panel. However, 16 out of 40 SNPs identified in the IGA transcriptome assembly were called correctly based on KASPar assay. Although the remaining 24 putative SNPs showed polymorphism between lines, they had low coverage (<10 reads) and did not meet our filtering criteria (at least 20 reads), therefore were not included in our final SNP dataset from the IGA transcriptome assembly.

## Discussion

We report on two transcriptome assemblies of pepper, the first is based on Sanger-EST sequences was used in the pepper GeneChip® project [11]. The second is based on a collection of transcriptomes of three pepper lines that were sequenced by IGA technology. The majority of pepper EST sequences that were used in the current project had been first assembled by Kim et al. (2008), in which they had assembled 22,011 unigenes with an average consensus sequences length of 1,688 bp. However, in order to construct the pepper GeneChip microarray prior to the Kim publication, we added all pepper sequences and resources that were available at the time (2006) of the assembly. In addition to *C. annuum* EST sequences from Korean F_1_ hybrid of Bukang, we added >700 sequences from other *C. annuum* cultivars and other pepper species such as *C. baccatum*, *C. frutescens* and *C. chinense*. We added pepper genomic and mRNA sequences from GenBank and COS marker sequences from Solanaceae Genome Network (SGN) and UC Davis [7]. We used a combination of several in-house scripts and CAP3 to make our assembly, while Kim et al. took a different approach to make the assembly. Regardless of the methods used to assemble EST sequences, the database that Kim et al. has created is useful *per se* to query for the sequence information and find annotation of each contig. We have enhanced the information for Sanger pepper ESTs by mining and validating a subset of SNPs from this assembly. We have also leveraged the information to develop an Affymetrix tiling array to construct two ultra-saturated genetic maps of pepper [27] and to evaluate genetic diversity in pepper breeding germplasm [11]. Overall, we were able to map >17,500 unigenes representing over 3,000 genetic bins of pepper [27]. In the second pepper assembly we attempted to capture as many transcribed genes as possible by collecting tissues from three different genotypes (than Bukang) in different developmental stages. Recently a transcriptome assembly of two pepper parental lines (CM334 and Taean) and their hybrid line (TF68) was carried out by Lu et al. [22,23]. Lu et al. used the GS-454 FLX Titanium (Roche, Mannheim, Germany) to sequence mRNA that was collected from fruits of greenhouse-grown peppers. The pepper land race, CM334, in the Lu et al. study was the same land race that we used, but they sequenced it by Roche 454 system and sampled fewer tissues. Furthermore, we normalized our libraries prior to sequencing. Using GS *de novo* assembling software (Newbler) they were able to assemble 25,597 (N50=911), 29,335 (N50=898) and 33,530 (N50=884) contigs in each of CM334, Taean and TF68, respectively. Functional annotation of these contigs was performed by FunCat [28], by which it was determined that the majority of contigs were involved in proteins with binding function, regulation and metabolism. These results are similar to our functional annotation. The Capsicum transcriptome database, a most recent study of pepper transcriptomes, was recently introduced by Góngora-Castillo et al. [24]. Using Sanger and GS-pyrosequencing technologies they sequenced thirty-three cDNA libraries of *C. annuum* var. Sonora Anaheim and *C. annuum* var. Serrano Tampiqueño. Finally, creating a hybrid assembly of Sanger-EST sequences and GS-pyrosequencing using the 454 Newbler program was made using over 1.9 M 454 reads and Sanger-EST sequences. This assembly consists of 32,314 contigs with N50 of 631 and contig length ranging from 100–3,033 nt. The number of contigs of their assembly was close to our Sanger-EST assembly, as well as the three pepper assemblies reported by Lu et al. [22,23]. However, the number of contigs might be slightly over estimated because they took into account contigs with a minimum of 100 nt in length, whereas in our Sanger-EST assembly the smallest contig was 200 nt.

While the 454 system generates long sequences, it suffers from low sequence depth, which is the unique advantage of the IGA system. Roche 454 performs poorly over the homopolymer regions of the genome. While IGA performs better on those regions, it has the disadvantage of generating short reads. Therefore a hybrid assembly of long and short reads to resolve the shortfalls of both sequencing systems would improve the quality of assembly. In spite of using IGA technology alone by sequencing three lines of pepper and boosting the number and length of reads (currently up to 120 nt) per IGA lane, we were able to assemble >135 M nucleotides in our assembly, which is 26 times more than any previously reported assemblies. In addition to the number of bases assembled, the N50 of the transcriptome assembly of this study is twice that of assemblies that were made with pyrosequencing alone.

In the present study we also annotated the two assemblies of pepper transcriptomes. According to the percentage of annotated contigs, 65% of the Sanger-EST assembly contigs and 35% of the IGA transcriptome assembly contigs were annotated. There are a number of reasons for the lower percentage of annotation of the IGA transcriptome assembly; one is that there were more novel sequences in the IGA transcriptome assembly compared to the Sanger-EST assembly. These new sequences did not have any hit in the GenBank, and as a result the number of sequences that were not annotated increased. Contig length also contributes to lower annotation. Since there were relatively more short contigs in the IGA transcriptome assembly than the Sanger-EST assembly, the percent of annotated sequences was lower. Also, during the Sanger sequencing procedure there is a cloning step involved in library construction, which favors selection for higher copy number transcripts, resulting in redundancy in annotated sequences and a lower number of unannotated sequences as well as poor sampling of single-copy sequences. Based on the number of annotated contigs our results for IGA analysis are similar to Lu et al. [22]. Considering the number of assembled nucleotides in contrast to the number of contigs, the present two assemblies were quite comparable, 70% in the IGA transcriptome assembly vs. 82% in the Sanger-EST assembly. In the Sanger-EST assembly 23% of the contigs or 17.5% of nucleotides did not align to any homologous sequences in the GenBank, therefore these sequences can be identified as potential novel transcripts or genes in pepper that were not previously characterized or simply were too short for conclusive annotation. Not surprising, the annotations of both assemblies presented here are very similar in terms of species distribution of top-hits. This is probably due to the bias in databases toward having more data for certain species that have been annotated better than the others. At the time of analysis tomato genome annotation was not available in GenBank databases which could be the reason as to why *S. lycopersicum* is not on the top of species hit list.

Another aspect of our study was to assign transcripts to different metabolic pathways. Generating KEGG maps and designating enzymes to different metabolic pathways is an effective way to identify candidate genes. In an ultra-saturated genetic map of pepper, contigs that are spanning a QTL can be further examined for their role in one or more metabolic pathways. Finding annotated contigs will then help to identify KEGG maps related to the enzymes and metabolites involved in the traits and further investigate their function in controlling traits.

One of our goals in this project was to develop markers that can readily be used in breeding programs. We presented here two sets of markers, SSR and SNP for genetic and breeding analyses in pepper. The putative SNPs that were discovered in the Sanger-EST assembly were internally validated by KASPar assays in a genotyping panel of 43 pepper lines and accessions. It is deemed to be very robust and reliable despite the lower sequence depth compared to SNPs that were discovered in the IGA transcriptome assembly. We also observed a comparable SNP frequency in both assemblies (1/2,798 bp vs.1/2,523 bp) indicating SNP frequency in pepper transcriptomes is plausibly consistent across methods and accessions used in different experiments. Coincidently, the polymorphism among three diverse lines, CM334, Early Jalapeño and Maor [11], and those within the F_1_-hybrid of Bukang was similar.

## Conclusions

There was a great need to generate an abundant number of molecular markers for breeding programs of pepper. To that end, assembling transcriptomes seemed very promising in the identification of thousands of high-quality markers before a pepper genome sequence becomes available. As a result of our efforts, the generated markers are currently being used in genetic mapping and QTL analyses by different groups around the globe. In order to have a better understanding of the assembled sequences and to identify candidate genes underlying QTLs, we also annotated the contigs of Sanger-EST and RNAseq assemblies. These and other information have been curated in a database that we have dedicated for pepper GeneChip project (see Data Access). Nevertheless, the main task still will remain to sequence the pepper genome and to use the available genetic resources to develop new pepper varieties with higher yields, better flavors and more resistance to biotic as well as abiotic stresses.

## Data access

The raw data are publically available through The NCBI Sequence Read Archive (SRA) under accession No. SRA052314.2 and the trimmed reads submitted to The European Nucleotide Archive (ENA) under study number ERP001411. The SNPs were submitted to database of Single Nucleotide Polymorphisms (dbSNP). Bethesda (MD): National Center for Biotechnology Information, National Library of Medicine. dbSNP accession:[ss523750580 – ss524344808], (dbSNP Build ID: B138), available from the NCBI SNP database website on the next build in December 2012. The IGA transcriptome assembly was submitted to NCBI transcriptome shotgun assembly database (TSA) under BioProject No. PRJNA163071 and TSA accession numbers JW05245 - JW111875. Both assemblies, annotations, SNPs, SSRs and other information are also available at https://pepper.ucdavis.edu/public/data.php.

## Methods

### Assembly of pepper Sanger-EST sequences

#### Source of Sanger-EST assembly sequences

Pepper sequences were obtained from two sources. A total of 115,787 EST sequences from 21 cDNA libraries (Korean GenePool database) of an F_1_ hybrid variety, Bukang, were kindly provided by Dr. Doil Choi (Korean Research Institute of Bioscience & Biotechnology (KRIBB), now at Seoul National University) [22]. These sequences were combined with other sequences from GenBank (in 2006), trimmed and passed through quality assessments to be used in assembly (see below). Tissue collection and cDNA library construction and Sanger sequencing has been described elsewhere [22]. GenBank Sequences (in 2006) included ESTs (31,495), mRNAs (515) and genomic sequences (464). Of these 21,590 were from KRIBB.

#### Preparation of sequences for assembly

To remove the redundant EST sequences from the GenBank collection, any sequence with an identical ID to the KRIBB collection was removed to obtain a non-redundant set of sequences. Genbank mRNA sequences were directly used for assembly. We identified two types of genomic sequences from the GenBank collection, annotated (218) and unannotated (246) sequences. The exon and intron regions of annotated sequences were known. Therefore we simply split out the introns to obtain the exonic sequences. In the case of unannotated sequences, the basic local alignment tool (BLASTX cutoff value = 1e^-20^) was used to search against plant reference genes to extract coding regions. The KRIBB sequences were merged with the processed GenBank sequences in the next step. The merged data set was further checked for regions containing low complexity sequences or vector sequences using custom made Python, TCL and Perl scripts that can be accessed from “atgc tools” website.

#### Clustering and assembly of pepper Sanger-EST sequences

CAP3 software [29] was used for assembling the sequences with overlap length cutoff of 100 and overlap percent identity cutoff of 90. Visualization and analysis of DNA sequences alignments generated by CAP3 (to detect and validate polymorphic sites either SNPs or InDels) was carried out using custom made “atgc-tools/align” scripts. These alignments and consensus sequences can be accessed through the pepper GeneChip website.

### Assembly of pepper IGA transcriptomes reads

#### Plant materials and library construction

The seed of three pepper (*C. annuum)* lines ‘CM334,’ ‘Maor’ and ‘Early Jalapeño’ (EJ) were planted in the greenhouses of the Department of Plant Sciences at UC Davis under standard conditions for *Capsicum*[30] until adult stage. Three cDNA libraries (one from each pepper variety) were prepared using pooled mRNA that was independently extracted from seven tissues: root, young leaf, 5, 10, and 20 days post pollination developing fruit, breaker and ripe fruit using Qiagen RNeasy Mini Kit (Qiagen Valencia CA, USA) per the manufacture’s protocol. CM334 root tissue was inoculated with *Phytophthora capsici* to induce expression of resistance genes. Aliquots were quantified using a NanoDrop spectrophotometer (NanoDrop Wilmington, USA) and checked for quality by electrophoreses separation using Lonza FlashGel System FlashGel RNA Cassettes (Lonza Inc. Allendale, USA). Samples were pooled in equivalent concentration. For each pepper line, paired-end libraries were prepared following standard Illumina protocols (Paired-End DNA Sample Prep Kit). The libraries were sheared and 300–350 bp fragments were selected on gels. The libraries were normalized using double-stranded nuclease to digest high copy double‐stranded DNA during re‐association after denaturation and then prepared for sequencing as described by Illumina [31]. The cDNA libraries were sequenced using Illumina Genome Analyzer II (IGA) for 85 cycles per direction at the UC Davis Genome Center. One lane of paired-end pass and one lane of single pass were run for each of CM334 and Maor lines and two lanes of paired-end pass were run for Early Jalapeño.

#### De novo assembly of IGA reads

The IGA data went through our standard preprocessing pipeline, developed at UCD (ILLUPA, A. Kozik, Pers. comm.). The trimming stringency was based on a study that was carried out by Alex Kozik to trim Illumina short reads of lettuce [32]. The reads (sequences) were first trimmed to discard traces of adapters and primers that were added to cDNA during library preparation using “cutadapt” software. Under the normal trimming scheme we trimmed the 5’ and 3’ ends of the reads with quality scores of lower than 20 (or 0.01 probability of error), then we retained the reads between a minimum length of 40 nt and a maximum of 85 nt with no further trimming (full filtered length). Under a more stringent procedure we trimmed the full filtered length reads (from above 40–85 nt) more robustly by trimming 10 nt from 5’ end and 5 nt from 3’ end of each read (25–70 nt length). As a result we maintained the reads with a length between 25 nt and 70 nt.

Velvet (v 1.0.14) [20] and CLC Genomics Workbench (v 4.0.3) software packages were used to assemble the sequences. For each pepper genotype, a Velvet assembly with several *k-mers* (31, 35 and 41 Velvet hash setting) was performed using full length trimmed and 25–70 nt length trimmed data. DNA *K-mer* is synonymous to a word in our language. It is a short consecutive stretch of DNA that will be used in *de bruijn* graph as described elsewhere [20].

The results of all *k-mer* assemblies were combined with CAP3 to make a line-specific super assembly. In other words, for each pepper line we obtained six Velvet assemblies (3 *k-mers* settings by 2 sets of reads) that were combined with CAP3 software yielding a super assembly. In addition to Velvet assemblies, two iterations of assembly (one for normally trimmed reads and one for stringently trimmed reads) with CLC genomic workbench with default settings (Insertion/deletion cost = 3, mismatch cost = 2, 80% of read length with similarity of 90%) were carried out for each pepper genotype. The results of the combined Velvet assemblies (super assembly) and CLC assemblies were merged using CAP3 software to make the Mega assembly for each line. Once we generated three Mega assemblies (one per pepper genotype), we combined the Mega assemblies from each line by CAP3 software to obtain a pepper transcriptome Meta (IGA) assembly. A graphical presentation of the assembly procedure is depicted in [See Additional File 3: Figure S16]. The IGA transcriptome assembly was submitted to NCBI transcriptome shotgun assembly database (TSA) under BioProject No. PRJNA163071 and TSA accession numbers JW05245 - JW111875.

### GO annotation of the Sanger-EST and the IGA assemblies

The Blast2GO [26] software was used to annotate both assemblies. Blast2GO involves three main steps, 1) BLASTX of the nucleotide sequence against the non-redundant protein database (nr) of NCBI, 2) mapping, retrieving GO terms associated with the blast results, and 3) annotating GO terms associated with each query in order to relate the sequences to known protein function. Briefly, a BLASTX search of contig nucleotide sequences against the non-redundant protein database (nr) of NCBI was performed under the default settings of BLAST2GO and the BLAST expectation value of 1.0e^-3^ and maximum 20 hits, HSP length cutoff (default = 33) with low complexity filter on was used. The GO terms associated with each BLAST hit were retrieved (mapping step) and GO annotation assignment (annotation step) to the query sequences was carried out using the following annotation score parameters; E-Value Hit Filter (default=1.0E-6), Annotation Cut-Off (default =55), GO-Weight (default=5), Hsp-Hit Coverage Cut Off (default = 0). In addition, contig sequences were queried for conserved domains/motifs using InterProScan, an on-line sequence search plug-in within the BLAST2GO program with all 13 applications selected before run and the resulting GO terms were merged with the GO term results from the annotation step of Blast2GO. KEGG maps for more than 130 metabolic pathways were generated with the KEGG extension of Blast2GO.

### Identification of SNPs in the Sanger-EST and the IGA assemblies

#### Sanger-EST assembly SNPs

In order to discover putative SNPs in the Sanger-EST assembly, the output files of CAP3 were used in the pipeline of SNP discovery (Alex Kozik, Pers. Comm.). In this method only contigs that are the results of assembling a minimum of two ESTs can be interrogated for the existence of putative SNPs. A total of 18,226 unigenes in the Sanger-EST assembly were singletons. As a result only 12,970 out of 31,196 unigenes were surveyed for SNPs. In Kozik’s pipeline, the EST sequences first align (map) to their corresponding consensus sequences. Second, at each position of consensus sequence the program searches the pileup of EST sequences for base changes among sequences. In the last step, the program outputs a list of contigs and positions where differences were found. A separate filtering step was carried out by a Perl script to select the SNPs with minimum depth of 2 for each SNP allele, 50 bp from the start or the end of a contig. If the two SNPs were in the vicinity of 50 bp from each other only the one with higher coverage was selected.

#### IGA transcriptome assembly SNPs

Assembling transcriptomes of three pepper lines enabled us to map all the IGA reads back to the assembly and to determine the putative SNPs. BWA [33], SAMtools, and in-house Perl scripts were used to call the SNPs. First we mapped all the short reads of each line separately to the assembly using BWA aligner to generate 3 BAM files. Using the SAMtools pileup command the variable positions (SNPs) were determined between the consensus pepper assembly and each line. The BAM files were also merged by SAMtools [34] and polymorphism were determined between the merged files and assembly. Custom written Perl scripts were used to generate a genotype table where we could line up the consensus assembly with genotype call for all three pepper lines. A position was called a putative SNP if two out of three pepper accessions/lines had the same homozygous allele (minimum depth of 10 reads), but different from the third homozygous accession. For instance, if CM334 and Maor were rendering a G allele at a given position and Early Jalapeño was carrying a C allele at the same position, then the position was called a putative SNP. In cases where the position of a SNP could not be unequivocally determined as described above then that position was called a heterozygote. The putative SNPs were then filtered against intron-exon junction positions using the command line version of Intron Finder software at Sol Genomics Network (SGN) website. The filtered putative SNPs were set to be at least 50 bp from intron/exon splice junctions as well as adjacent SNPs and heterozygote positions.

### Validation of SNPs in the Sanger-EST assembly

In order to validate the *in silico* SNPs from the Sanger-EST assembly, 50 nucleotides from either side of 142 SNPs, (40 of which corresponded to SPP markers from a Pepper diversity panel [11]) were extracted from each contig. Sequences were sent to KBiosciences to develop KASPar assays. The assays were run by KBioscience on a diversity panel of 47 lines and cultivars and the data was visualized by KBioscience SNP viewer software and further analyzed with Microsoft Excel.

### Validation of SNPs in the IGA transcriptome assembly

The three pepper lines that were used for the IGA transcriptome assembly were also included in the genotyping panel that was surveyed for SNPs by KASPar assay. We used BLASTN to find near identical sequences of the IGA transcriptome assembly to 101 bases flanking each SNP (50 nt each side) that was used in the KASPar assay. If a hit was found with 95% sequence similarity and e^-20^ expectation value, then we investigated the possibility of calling the same SNP in the IGA transcriptome assembly by scanning the list of IGA transcriptome based SNPs.

### *In silico* identification of SSRs in Sanger-EST and IGA transcriptome assemblies

The assembled sequences were used to identify signatures of SSRs. FASTA files containing all the assembled sequences were used as an input file in MISA Perl script to specify the minimum number of the following repeats for microsatellites (unit size/minimum number of repeats): (2/6) (3/5) (4/5) (5/5) (6/5). MISA has the capability of predicting perfect (SSRs with no interruption) and compound SSRs (SSRs with a spacer sequence). The variable to specify the maximum length of the spacer sequence was set as 100bp in the MISA setup file.

### Primer design for SSRs

The PRIMER3 software [35] was used to design forward and reverse primers flanking the SSR containing sequence. An accompanying Perl script of MISA software (*p3_in.pl*) was used to make the input file for PRIMER3. A second accompanying Perl script of MISA software (*p3_out.pl*) was used to parse the output file of PRIMER3 into a user friendly output. The target amplicon size was set as 100-300bp, with optimal annealing primer temperature of 60 °C and optimal primer length as 20 nucleotides.

## Abbreviations and URLs

454 Life Sciences: http://www.454.com; ABySS: http://www.bcgsc.ca/platform/bioinfo/software/abyss; Alexander Kozik SNP discovery pipeline: http://cgpdb.ucdavis.edu/SNP_Discovery_CDS/; Alexander Kozik Illupa pipeline: http://code.google.com/p/atgc-illumina/downloads/list; atgc-Tool website: http://code.google.com/p/atgc-tools/; atgc-Tool/align website: http://code.google.com/p/atgc-align/; CAP3 Software: http://pbil.univ-lyon1.fr/cap3.php; CLC Genomics Workbench: http://www.clcbio.com/; Cutadapt software: http://code.google.com/p/cutadapt/; European Nucleotide Archive (ENA): http://www.ebi.ac.uk/ena/; GenBank (NCBI): http://www.ncbi.nlm.nih.gov/; Illumina GoldenGate Assay/Illumina Technology: http://www.illumina.com/; InterProScan: http://www.ebi.ac.uk/Tools/pfa/iprscan/; KBioscience: http://www.kbioscience.co.uk/; KBioscience SNP Viewer Software: http://www.kbioscience.co.uk/software/Software_intro.html; KEGG Maps: http://www.genome.jp/kegg/pathway.html; Korean GenePool database: http://genepool.kribb.re.kr; Korea Research Institute of Bioscience and Biotechnology: http://www.kribb.re.kr/eng/; The NCBI Sequence Read Archive (SRA): http://www.ncbi.nlm.nih.gov/Traces/sra; The NCBI SNP data base: http://www.ncbi.nlm.nih.gov/SNP/; MISA Perl Script: http://pgrc.ipk-gatersleben.de/misa/; Newbler: http://454.com/products/analysis-software/index.asp; Pepper GeneChip Website: https://www.pepper.ucdavis.edu/; Sequenom: http://www.sequenom.com/; SOAPdenovo: http://soap.genomics.org.cn/; SOLiD: http://www.appliedbiosystems.com/absite/us/en/home/applications-technologies/solid-next-generation-sequencing.html; Sol Genomics Network: http://solgenomics.net/; Transcriptome shotgun assembly database (TSA): http://www.ncbi.nlm.nih.gov/genbank/tsa; Velvet Sequence Assembler: http://www.ebi.ac.uk/~zerbino/Velvet/.

## Competing interests

This project was supported by an award from the University of California UC Discovery Program bio06-10565 with matching funds from Rijk Zwaan BV, Enza Zaden BV, DeRuiter Seeds B.V., Nunhems USA, Seminis Vegetable Seeds, Inc., Syngenta Seeds, Inc. and Vilmorin Co. The funders had no role in study design, data collection and analysis, decision to publish, or preparation of the manuscript. Funds were provided directly to the University of California Davis. Authors have no competing interests for this research.

## Authors' contributions

HA wrote the manuscript and performed IGA transcriptome assembly, SNP and SSR discovery. TAH, planted peppers, collected tissues, prepared DNA for KASPar assay and analyzed the KASPar assay data, read and edited the manuscript. KS, developed the mRNA library for IGA sequencing. SCR, developed SNP discovery pipeline in the transcriptome and designed KASpar assays. AK, co-designed the project and developed the SNP detection pipeline of EST assembly. JY, processed and assembled Sanger ESTs sequences. AVD, envisioned, realized the project and supervised the group. All authors read and edited the manuscript. All authors read and approved the final manuscript.

## Supplementary Material

Additional file 1 **Ashrafi et al. 2012 Pepper Annotation Supp 05072012.** A Microsoft-Word 2007 file with 16 figures comparing the results of Blast2GO for GeneChip (Sanger-EST) and transcriptome assemblies of pepper as well as the IGA transcriptome assembly procedure flow chart. Click here for file

Additional file 2 **Ashrafi et al. Pepper Assembly Supp 05072012.** A Microsoft-Excel 2007 file with 2 tables (worksheet) corresponding to Annotation results of the Sanger-EST and IGA transcriptome assemblies of pepper. Click here for file

Additional file 3 **Ashrafi et al. Pepper Assembly Supp 05072012.** A Microsoft-Excel 2007 file with 13 tables corresponding to SSR and SNP lists identified in both the Sanger-EST and IGA transcriptome assemblies of pepper. It also includes identified SSR motifs and list of diversity panel (adapted from Hill et al. [11]). Click here for file
