# Supplementary material for: De novo assembly of the pepper transcriptome (Capsicum annuum): a benchmark for in silico discovery of SNPs, SSRs and candidate genes
Source: BMC Genomics. 2012 Oct 30;13:571. doi: 10.1186/1471-2164-13-571 (PMC3545863; doi:10.1186/1471-2164-13-571)
Supplement: Additional file 1 — Ashrafi et al. 2012 Pepper Annotation Supp 05072012. A Microsoft-Word 2007 file with 16 figures comparing the results of Blast2GO for GeneChip (Sanger-EST) and transcriptome assemblies of pepper as well as the IGA transcriptome assembly procedure flow chart. [file 1471-2164-13-571-S1.docx]

### Additional_file_3_Ashrafi_et_al_2012_Pepper_Annotation_Supp_05072012.docx

A Microsoft-Word 2007 file with 16 figures comparing the results of Blast2GO for GeneChip (Sanger-EST) and transcriptome assemblies of pepper as well as the IGA transcriptome assembly procedure flow chart.

***De novo* assembly of the pepper transcriptome (*Capsicum annuum*): a benchmark for *in silico* discovery of SNPs, SSRs and candidate genes**

AUTHORS:

Hamid Ashrafi, Theresa Hill, Kevin Stoffel, Alexander Kozik, Jiqiang Yao, Sebastian Reyes Chin-Wo and Allen Van Deynze


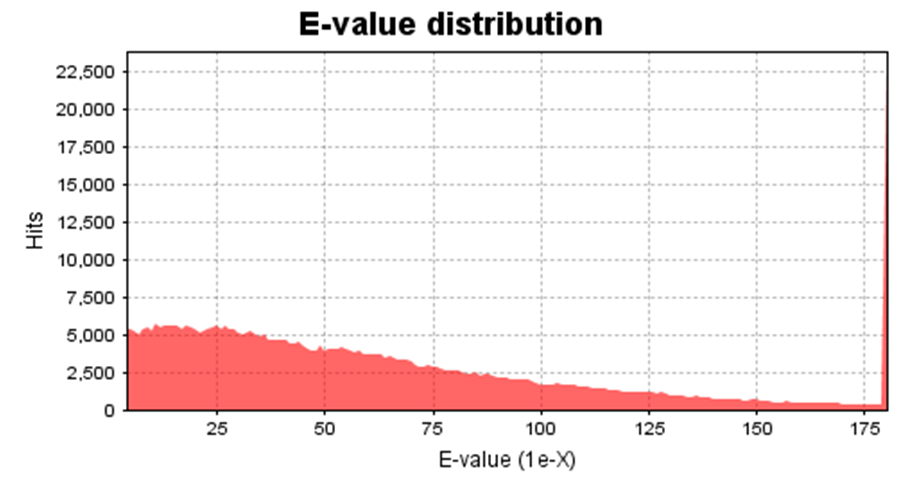

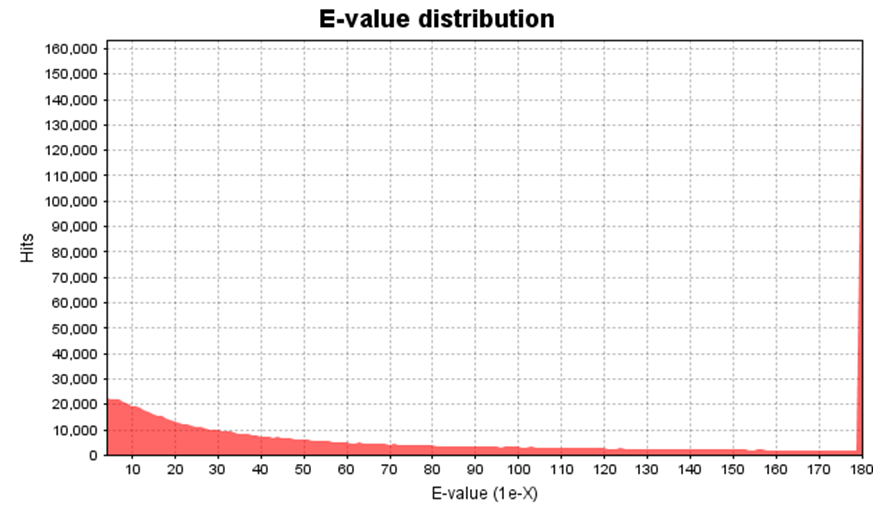


a

b

**Supplement Figure 1.** Distribution of E-Values of BLASTX of a) the Sanger-EST unigenes b) IGA transcriptome contigs

a

b


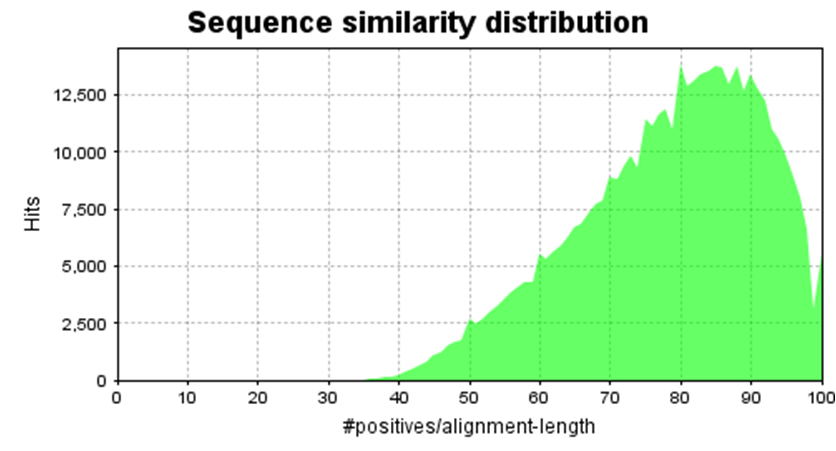

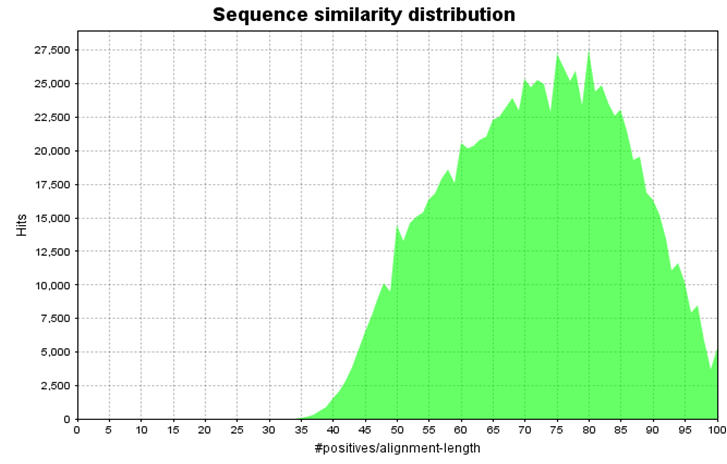


**Supplement Figure 2.** Percent Similarity of assembly sequences with sequences in the GenBank a) Sanger-EST unigenes b) IGA transcriptome contigs. Similarity is computed of each query-hot pair as the sum of similarity values for all matching HSPs

a

b


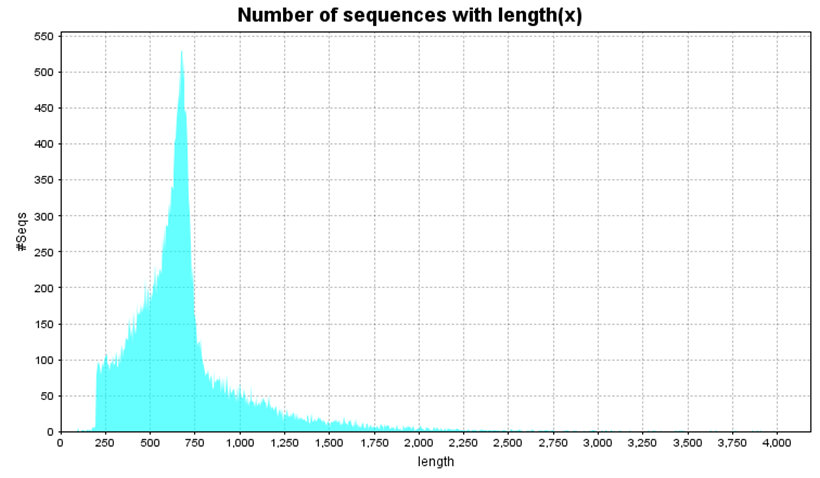

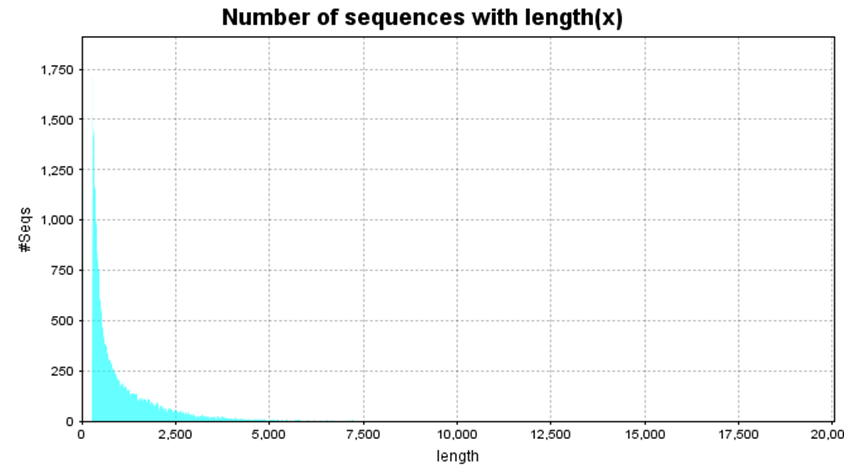


**Supplement Figure 3.**  Length vs number of sequences in a) Sanger-EST unigenes b) IGA transcriptome contigs.

a

b


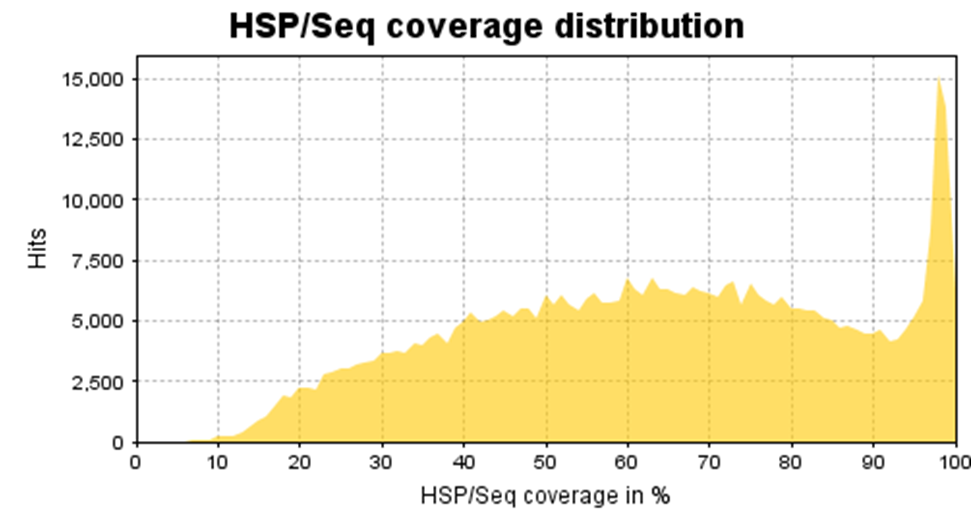

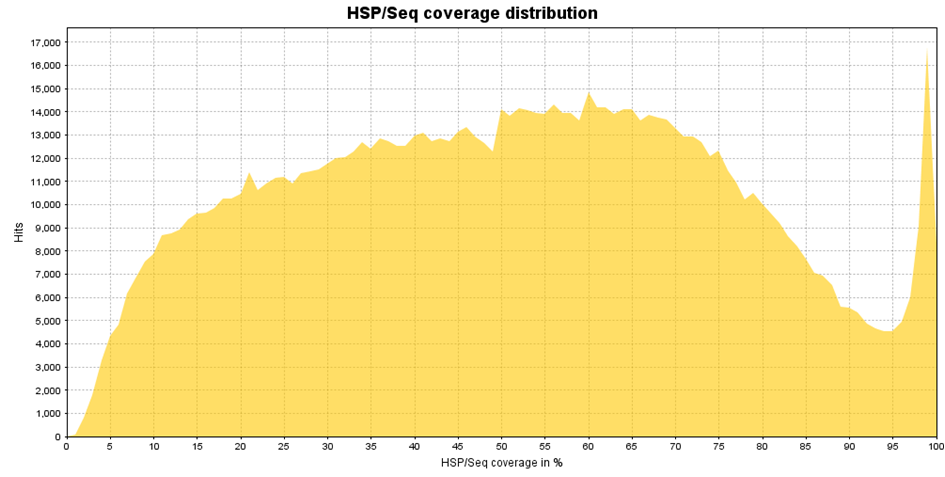


**Supplement Figure 4.**  High-scoring segment pairs (HSP) per sequence coverage a) Sanger-EST unigenes b) IGA transcriptome contigs.

**Supplement Figure 5.**  Evidence code distribution^[[1]](#footnote-1)^ sequences depicts the inference about the annotation. For instance IEA is inferred from electronic assay, or IDA inferred from direct assay. a) Sanger-EST unigenes b) IGA transcriptome contigs.

a

b


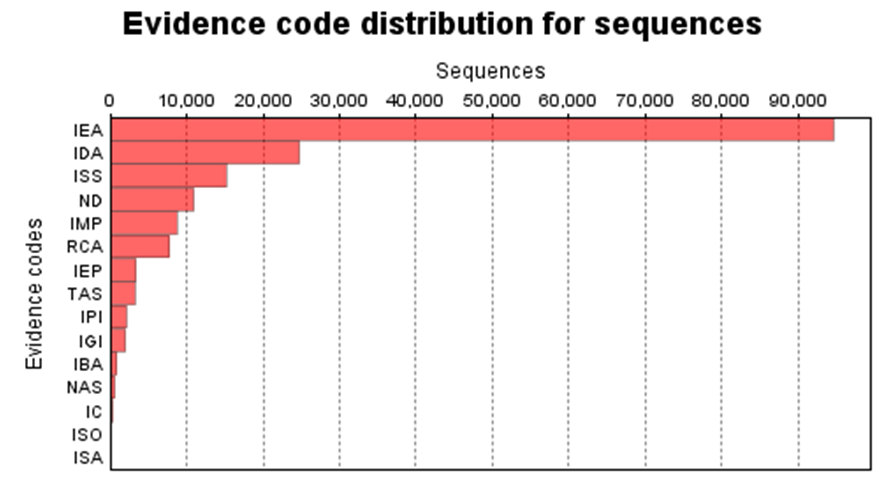

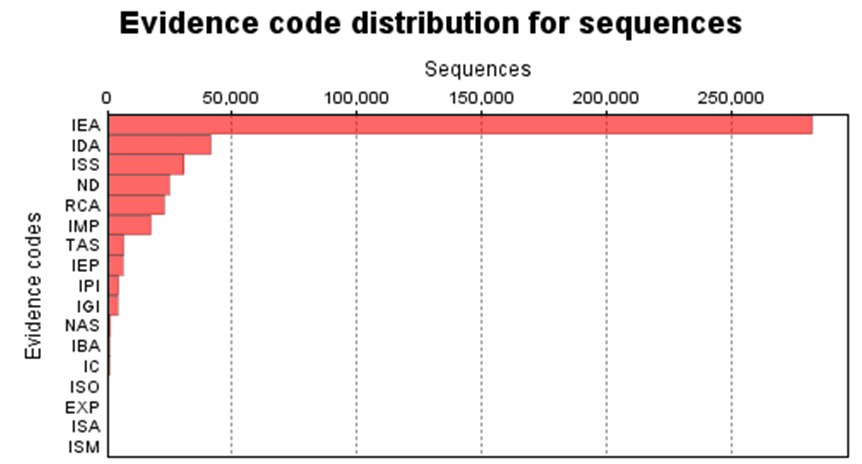


a

b


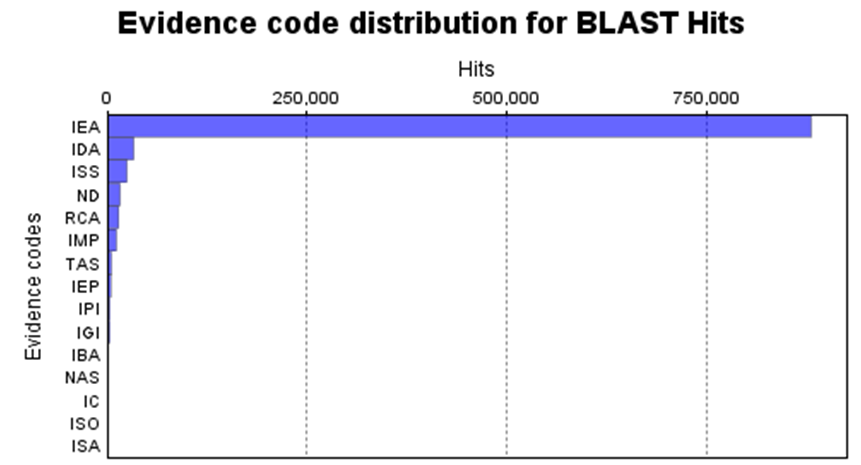

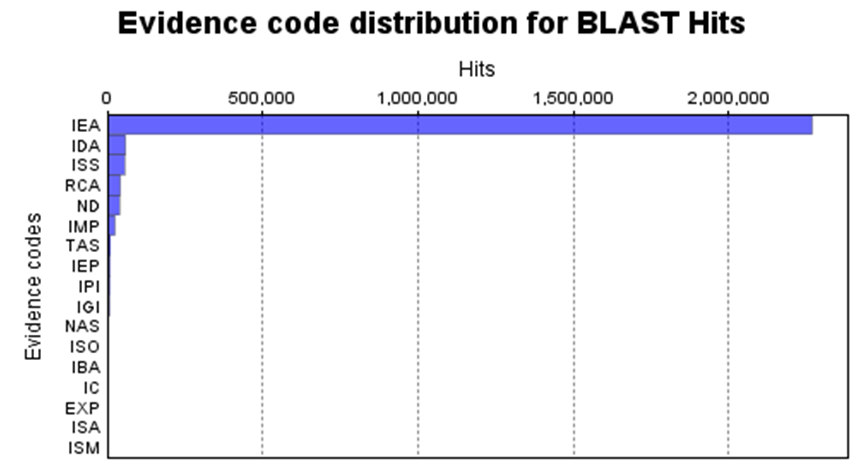


**Supplement Figure 6.**  Evidence code distribution for BLAST hits depicts the inference about the annotation. For instance IEA is inferred from electronic assay, or IDA inferred from direct assay. a) Sanger-EST unigenes b) IGA transcriptome contigs.

a

b


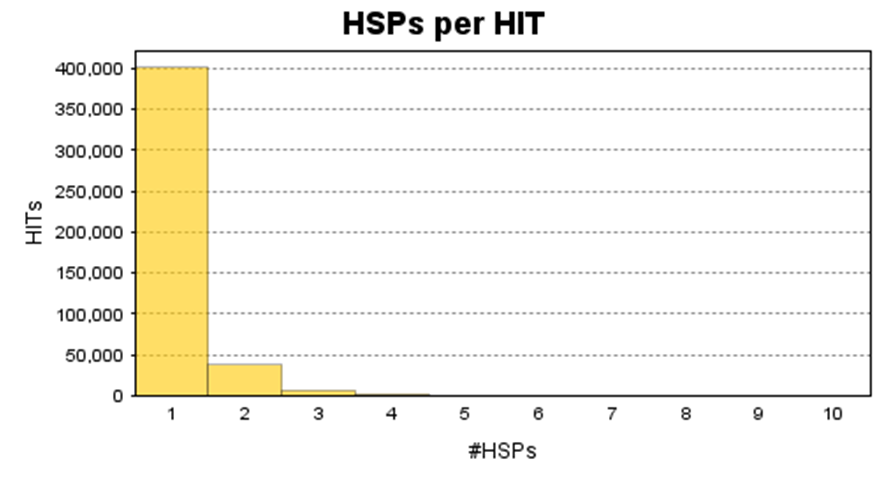

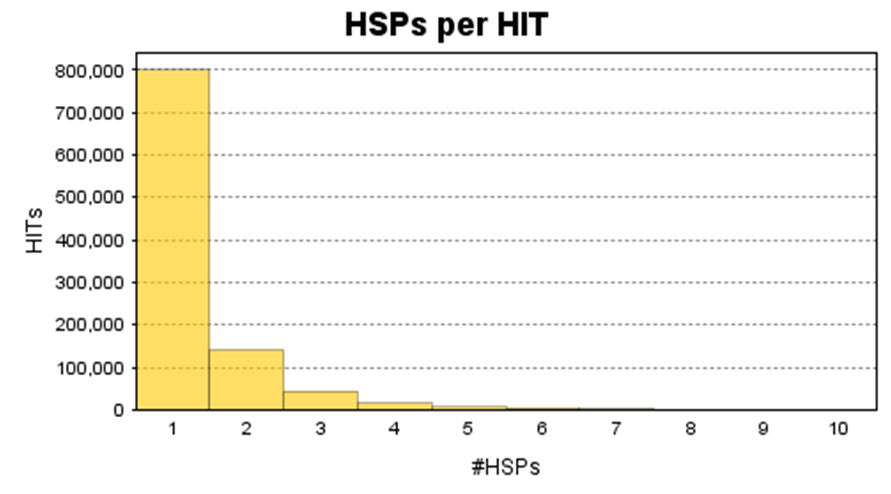


**Supplement Figure 7.** Number of high similarity pairs per BLAST hit **a)** Sanger-EST unigenes **b)** IGA transcriptome contigs.

a

b


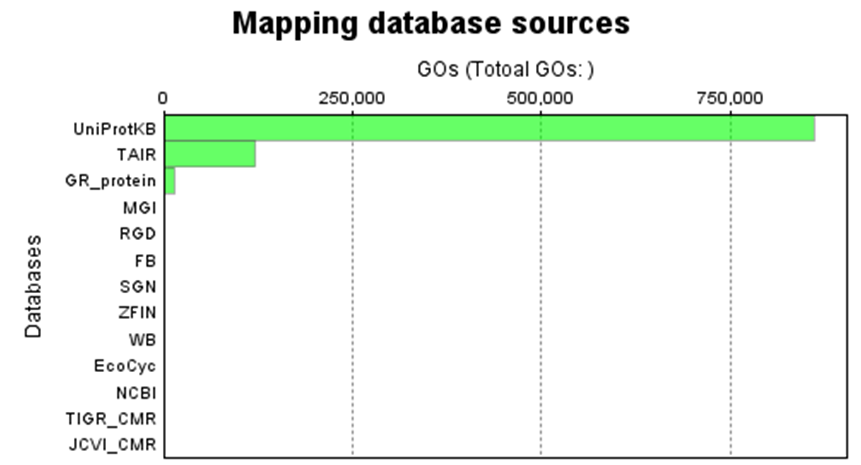

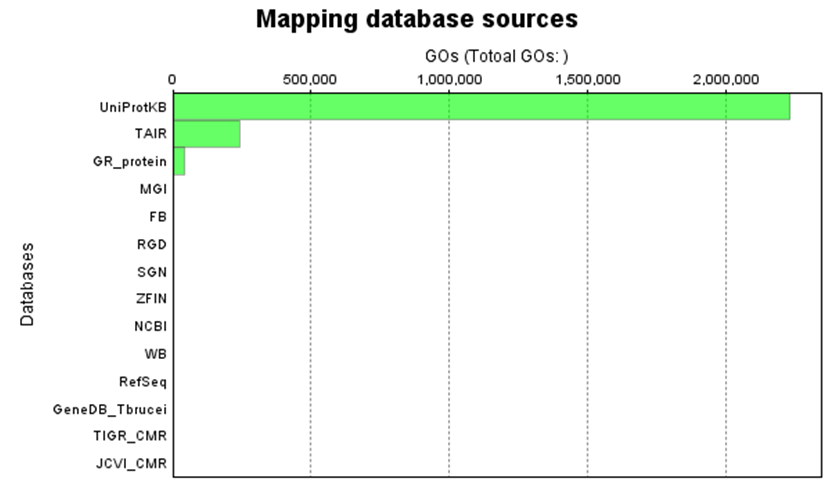


**Supplement Figure 8.** Database resources that were used for mapping step of BLAST2GO **a)** Sanger-EST unigenes **b)** IGA transcriptome contigs.

**a**

**b**

**Supplement Figure 9.** Number of GO terms per contigs. **a)** On average (weighted average) 5 GO terms was mapped to 19,966 (64%) contigs of Sanger-EST assembly. **b)** on average (weighted average) between 5 GO terms was mapped to 37,000 (30%) contigs of IGA transcriptome assembly.


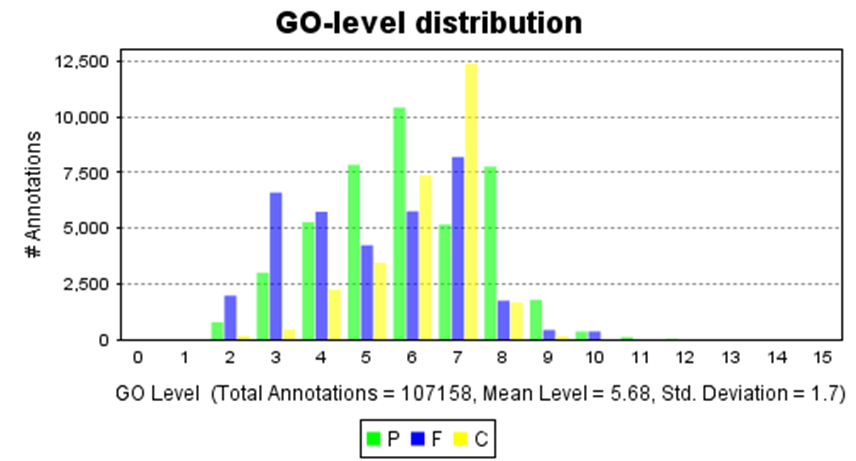

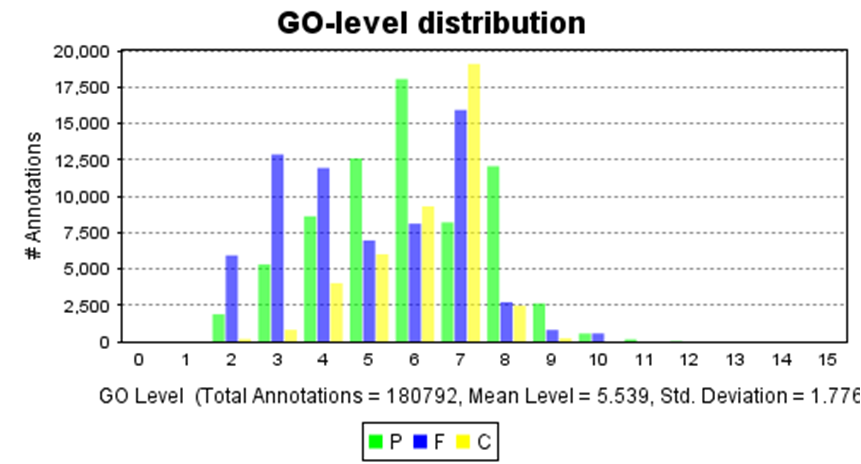


a

b

**Supplement Figure 10.** Number of annotations at each GO level. P for Biological Processes, F for Molecular Function and C stands for Cellular components. **a)** Sanger-EST unigenes **b)** IGA transcriptome contigs.

.

**a**

**b**

**c**

**Supplement Figure 11.** Direct GO count graphs depicting, **a)** Biological processes **b)** Cellular components and **c)** Molecular functions in the Sanger-EST assembly.

**a**

**b**

**c**

**Supplement Figure 12** The Direct GO count graphs depicting, **a)** Biological processes **b)** Cellular components **c)** Molecular functions in the IGA transcriptome assembly.

a

b


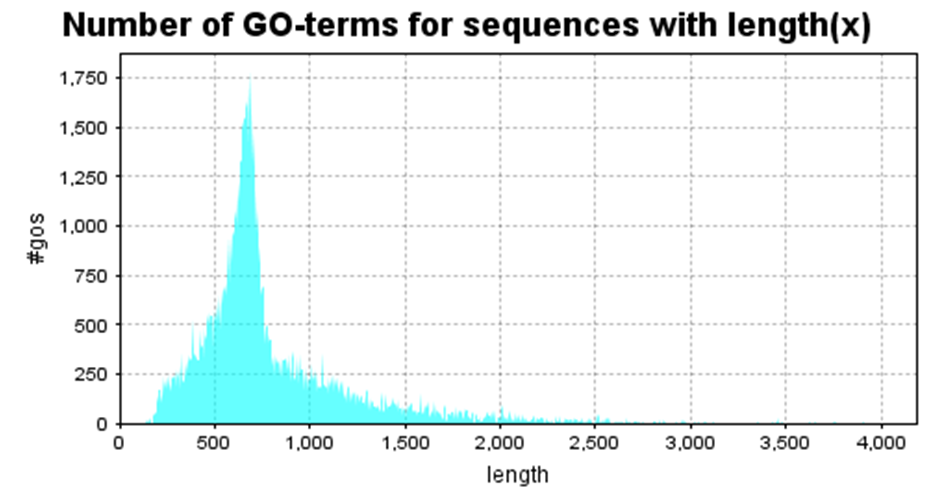

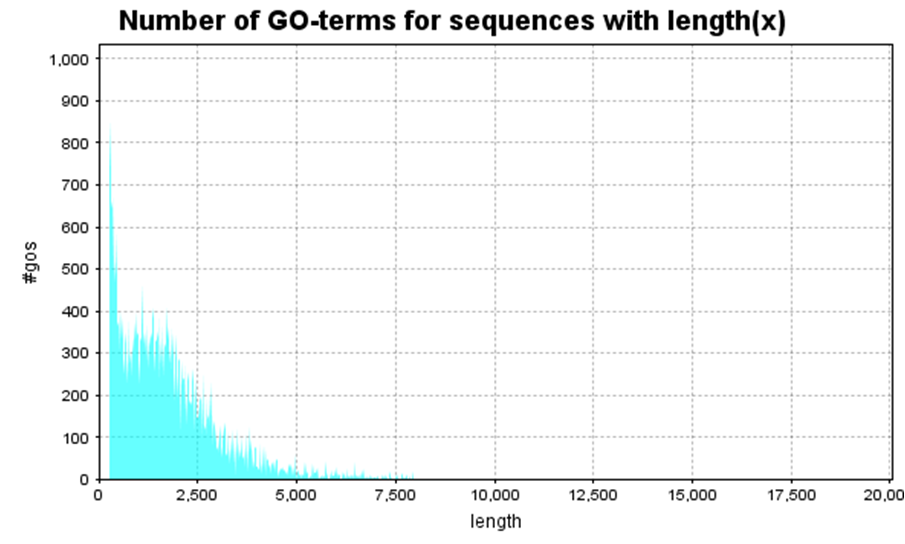


**Supplement Figure 13.** The relationship between number of Go terms and length of sequences. **a)** Sanger-EST unigenes **b)** IGA transcriptome contigs.

a

b


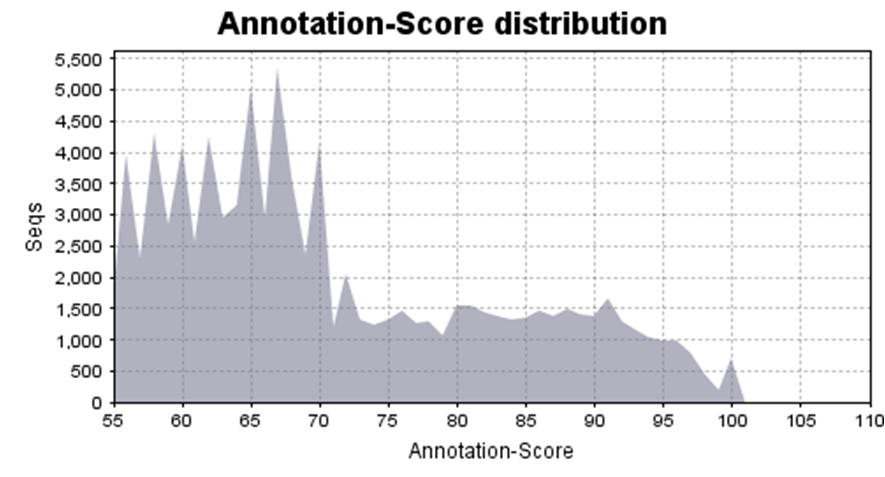

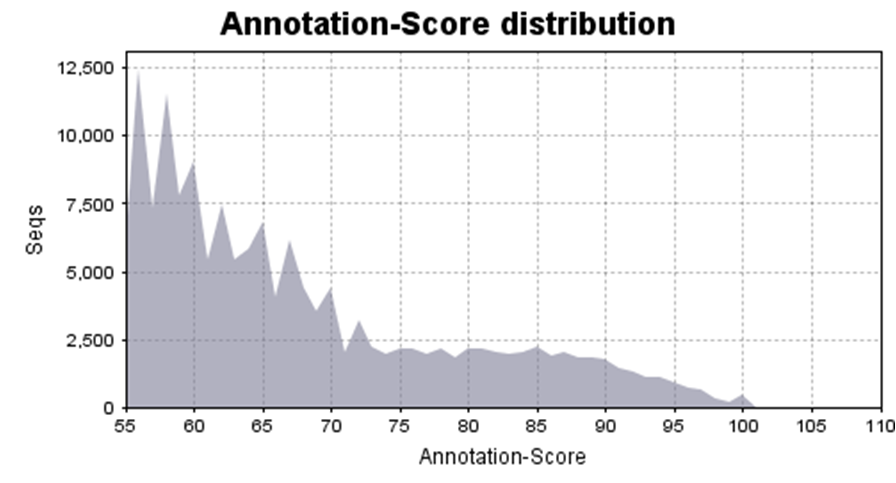


**Supplement Figure 14.** Distribution of annotation score vs. number of sequences **a)** Sanger-EST unigenes **b)** IGA transcriptome contigs.

a

b


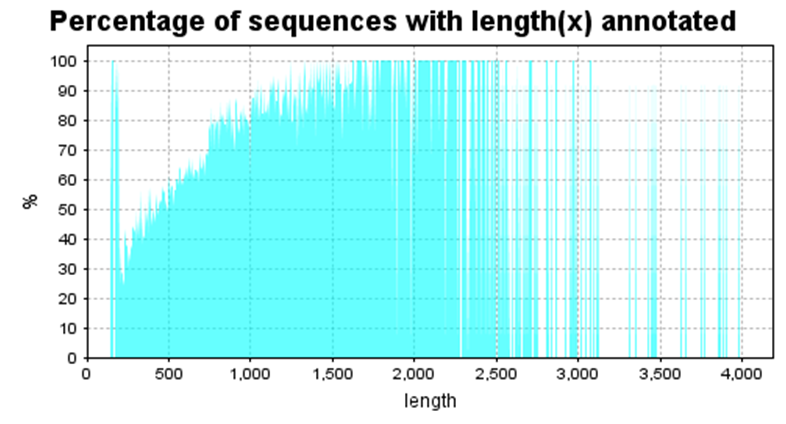

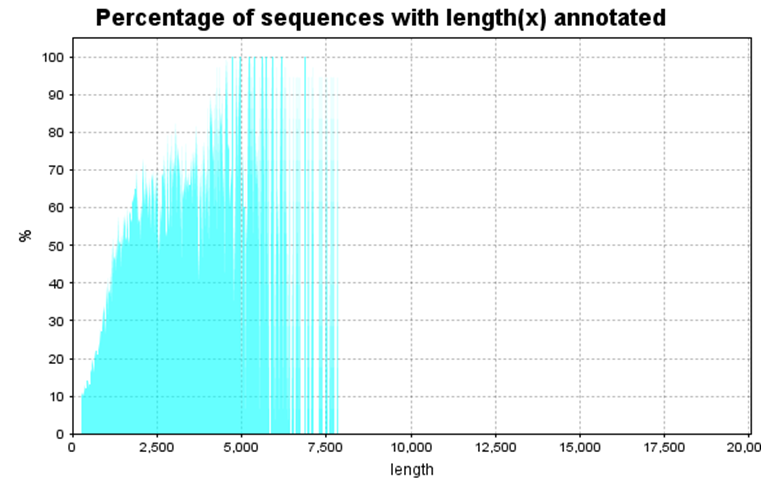


**Supplement Figure 15.** The relationship between length of sequence and annotation **a)** Sanger-EST unigenes **b)** IGA transcriptome contigs.

**
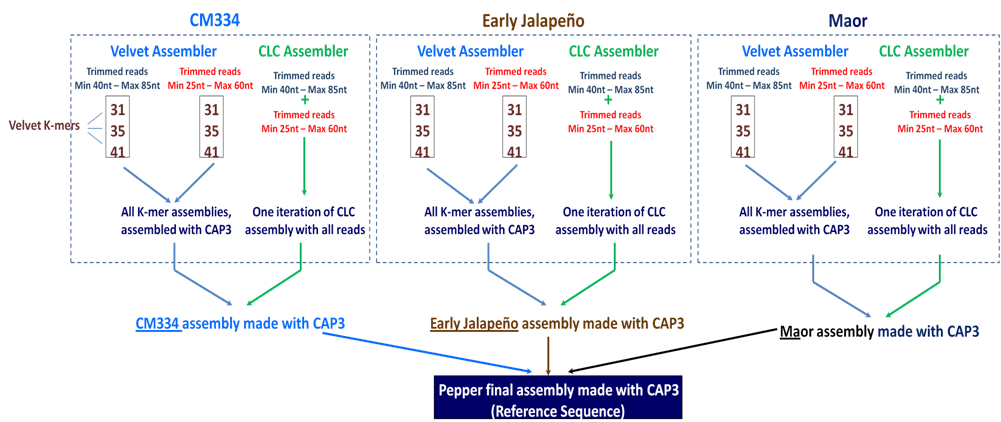
**

**Supplement Figure 16**: A flow chart of steps taken to assemble pepper IGA reads. Super assembly comprises of the combined assembly of Velvet K-mers or CLC workbench iterations (within each square box two super assemblies). The assembly of each super assembly is depicted by different colors to show Mega assemblies (immediately below each box). The Mega assemblies were combined to make Meta assembly (navy blue box marked as reference sequence).

1. Once mapping has been completed, the user can check the distribution of evidence codes in the recovered GO terms and the original database sources of annotations. These charts give an indication of suitable values for B2G annotation parameters. For example, when a good overall level of sequence similarity is obtained for the dataset, the default annotation cutoff value could be raised to improve annotation accuracy. Similarly, if evidence code charts indicate a low representation of experimentally derived GOs, the user might choose to increase the weight given to annotations. After the final annotation step, new charts show the distribution of annotated sequences, electronic the number of GOs per sequence, the number of sequences per GO, and the distribution of annotations per GO level, which jointly provide a general overview of the performance of the annotation procedure. [↑](#footnote-ref-1)
